# International qualitative case studies of system-level approaches to promote the development, adoption, and implementation of artificial intelligence in healthcare

**DOI:** 10.1093/jamia/ocag148

**Published:** 2026-09-08

**Authors:** Kathrin Cresswell, Hajar Mozaffar, Stuart Anderson, Xiao Yang, Sara Bea, Lucas Seuren, Robin Williams

**Affiliations:** Usher Institute, The University of Edinburgh, Edinburgh EH16 4UX, United Kingdom; Business School, The University of Edinburgh, Edinburgh EH8 9JS, United Kingdom; School of Informatics, The University of Edinburgh, Edinburgh EH8 9AB, United Kingdom; School of Informatics, The University of Edinburgh, Edinburgh EH8 9AB, United Kingdom; Institute for the Study of Science, Technology and Innovation, The University of Edinburgh, Edinburgh EH1 1LZ, United Kingdom; Usher Institute, The University of Edinburgh, Edinburgh EH16 4UX, United Kingdom; Usher Institute, The University of Edinburgh, Edinburgh EH16 4UX, United Kingdom; Institute for the Study of Science, Technology and Innovation, The University of Edinburgh, Edinburgh EH1 1LZ, United Kingdom

**Keywords:** artificial intelligence, scaling, evaluation

## Abstract

**Objectives:**

Artificial intelligence (AI) is increasingly being deployed in health-care settings, yet health systems remain at an early stage in developing approaches to scale effective applications. Much of current activity is characterized by early-stage use cases and pilot projects. In this study, we explored how different health systems are approaching the development, adoption, implementation, and scaling of AI in healthcare to identify transferable lessons for system-level change.

**Materials and Methods:**

We conducted 4 qualitative case studies of health systems and their approaches to scaling AI in healthcare: Catalonia, Norway, Singapore, and Queensland. Data were generated through documents, qualitative interviews and focus groups with strategic decision-makers, policymakers, and lead clinicians. The study explored participants’ perspectives on evolving strategies and needs, rationales for change, characteristics of approaches and technologies, expected and experienced benefits, lessons learned, perceived challenges, and elements considered transferable across contexts. Analysis was conducted in 2 stages: an initial within-case thematic analysis, followed by a cross-case analysis informed by the Technology, People, Organization, and Macroenvironment framework, while also allowing inductive themes to emerge.

**Results:**

The dataset consisted of 60 documents, 34 interviews, and 5 focus groups. We consulted a total of 50 different participants from case study sites across data collection activities. Our findings indicate that AI scaling trajectories in health-care systems are strongly shaped by existing digital strategies, historical and cultural contexts of digitalization, funding arrangements, and legacy technological infrastructures. Approaches to accelerating safe AI adoption at scale were characterized by efforts to orchestrate and coordinate change across multiple stakeholder groups with differing and evolving spheres of influence. Within this context, scaling was shaped by opportunities for experimentation, incentive structures, and the distribution of decision-making authority. Sustained use and wider spread of AI applications can be supported through ongoing post-deployment monitoring and the central collation and dissemination of emerging evidence. Crucially, these activities need to be embedded within a broader ecosystem that foregrounds learning, partnership, governance, and procurement as integral components of scaling.

**Conclusion:**

Our findings suggest that strategic decision-makers need to move beyond the conventional dichotomy of “top-down” and “bottom-up” approaches to scaling AI and instead conceptualize scaling as a process of multilevel orchestration.

## Introduction

Artificial Intelligence (AI)-based innovations promise to transform the quality, safety and efficiency of health and care systems, but their impact has consistently been constrained by difficulties in moving beyond isolated pilots to widespread, sustained use.^1–3^ Expectations of rapid and transformative uptake of AI tools have been challenged by early experiences of slow and difficult roll out.^4^ Implementation and validation are more costly than anticipated, generating robust evidence has been challenging, and the regulatory landscape is still evolving.

Although procuring centrally is extremely difficult, the currently prevailing model of ad hoc acquisition of individual tools by single provider organizations has also proved problematic. High implementation and evaluation costs have made it difficult for vendors to sustain profitability and also for provider organizations to establish compelling business cases. As a result, many early startups have struggled and ultimately exited the market.^5–8^

At the same time, AI products remain relatively immature, and there has been limited learning about how best to embed, optimize, and scale AI tools in real clinical settings.^9^ A key challenge going forward is therefore to develop institutional and technical arrangements for health systems to procure, validate, optimize, and conduct post-market surveillance of AI tools across multiple provider organizations.

The World Health Organization (WHO) defines scaling as “deliberate efforts to increase the impact of successfully tested health innovations so as to benefit more people and to foster policy and program development on a lasting basis.”^10^ However, while decades of research on diffusion of innovations and on scaling in public health and quality improvement have generated a rich body of conceptual frameworks, much of this literature has conceptualized scaling as a linear, stage-based process, typically focused on the replication of relatively stable interventions across organizational settings.^11–18^

Contemporary health systems are increasingly understood as complex adaptive systems in which spread and scale are socially mediated, context dependent, and shaped by dynamic interactions between technologies, users, organizations, and policy environments.^19^^,^^20^ In many health-care settings, decision-making authority is distributed across multiple organizations and stakeholder groups, creating polycentric systems characterized by multiple centers of decision-making and influence.^21^ In such environments, scaling requires orchestration, or the deliberate coordination and alignment of activities across multiple actors, organizations, and levels of the system.^22^ As a result, there is growing recognition that scaling in digitalization is not only about technical performance and implementation but also an ongoing process of adaptation, learning, and system change that must be actively facilitated and orchestrated.^23^

Within healthcare, several strands of scholarship have sought to move beyond linear adoption models by conceptualizing spread, scale-up, and sustainability as closely interlinked dimensions of a dynamic learning process (Table 1). Although the concepts of scale-up, spread, and sustainability are frequently used in the digital health literature, they are often applied inconsistently.

**Table 1.** Definitions of terms.^24^^,^^25^

**Scale-up** refers to deliberately expanding a technology, program, or intervention so that it is delivered to a larger population or in new organizational contexts, by building the infrastructure, workforce capability, and operational systems necessary for broader implementation beyond initial pilot or early adopter settings **Spread** is the process by which an innovation is taken up in different settings or organizations, often through informal or organic mechanisms (eg, peer networks, professional communities, shared learning) rather than central mandates, enabling replication of successful practice across diverse contexts **Sustainability** means the continued use and embedding of a technology, program, or practice over time, such that its benefits are maintained and the implementation becomes part of routine work, surviving changes in context, staff, or organizational priorities

Public health, quality improvement, and the learning health system literature increasingly emphasizes the coevolution of technologies and contexts; the centrality of organizational and professional work; the importance of supportive infrastructures, governance, and learning mechanisms; and the cultivation of communities of practice and knowledge exchange networks that allow innovations to spread and be adapted to suit local contexts and needs.^24–31^

Despite this, the literature on the scaling of AI-based interventions in health and care remains sparse, exacerbated by limited high-quality evaluations of adoption processes equipped to understand contextual variations and the speed at which technologies are evolving.^32–34^ AI technologies also introduce additional challenges: dependence on high-quality data and technology infrastructures, variability and opacity around performance impacting user trust, regulatory and ethical concerns, and the need for continuous model updating and re-validation.^35–37^

While there is a growing literature on AI adoption and implementation in healthcare, most studies focus on individual technologies, organizations, or implementation projects. Comparatively little attention has been paid to how health systems are attempting to coordinate, govern, evaluate, and support AI adoption at scale across multiple organizations. As health systems increasingly seek to move beyond pilots toward sustained and widespread use of AI, there is a need to understand emerging system-level approaches and the conditions under which they may be effective.

Consequently, it is critical to understand how health systems are navigating the scaling of AI to identify which approaches are effective in what contexts and how AI can be implemented and adopted in ways that maximize benefits while minimizing harms. We therefore sought to explore how different health systems are approaching the development, adoption, implementation, and scaling of AI in healthcare to identify transferable lessons for system-level change. Although the study was initially motivated by lessons for NHS England, the challenges associated with coordinating AI adoption across multiple stakeholders are shared by many health-care systems internationally, including those with more fragmented governance arrangements.

## Methods

We conducted a qualitative study using a series of case studies of different health systems to understand different approaches to AI adoption, implementation, and scaling.

Ethical approval was granted by the University of Edinburgh Medical School Research Ethics Committee (reference: 25-EMREC-012_NSA01) on February 5, 2025. We obtained written informed consent from all participants before their involvement in the research.

### Sampling

We undertook a scoping exercise to identify a diverse range of system-level approaches to promote the development, adoption, and implementation of AI in healthcare across different countries and regions. This initial phase focused on mature health systems that we judged would yield important insights into system-level approaches to scaling AI in healthcare. We prioritized jurisdictions with explicit AI strategies, established digital health infrastructures, and identifiable mechanisms for coordinating AI adoption and implementation across multiple provider organizations. Although the study was initially motivated by lessons for NHS England, we sought to identify approaches with broader relevance to health systems internationally. We identified 16 candidate health systems based on maximum variation in relation to geographical regions, information infrastructures, approaches to AI governance, and public-private partnerships. We focused on health systems and/or regions with a national AI strategy and on high-income settings as these were likely to be most comparable to NHS England. We also sought to sample health systems with similar health priorities including chronic disease management, and aging populations. Health systems were identified through a combination of web searches of the empirical and gray literature, as well as through consulting individuals from our existing international networks.

We then selected 6 sites for in-depth study and approached gatekeepers in each of these sites. Gatekeepers included senior policymakers, innovation leaders, academics, and digital transformation leaders who were identified through professional networks, published reports, and publicly available organizational information. Contacts in 2 sites did not respond to our invitation. We followed up these invitations a week after the initial invitation but still received no response. Our final 4 sites were Catalonia (Spain), Singapore, Queensland (Australia), and Norway. While we considered a range of potential case study sites, including jurisdictions with strong records of AI innovation, we prioritized settings where system-level approaches to coordinating AI adoption and scaling could be examined across multiple provider organizations and governance levels.

It is important to recognize that the health systems included in this study differ substantially in scale and organization from NHS England. System size appears to be a critical factor. Institutional arrangements that function effectively in jurisdictions with mid-sized populations (around 5 million people) may be easier to coordinate and sustain than those operating across much larger systems of 50 million or more.

In several cases examined, health system-level coordination takes place at the level of devolved or state administrations rather than across an entire nation. Catalonia, for example, is an autonomous community within Spain with significant self-governing powers and responsibility for health services through the Government of Catalonia (Generalitat de Catalunya).^38^ Similarly, Queensland operates within Australia’s federal system as a state with its own parliament and government responsible for organizing and delivering healthcare. In both settings, coordination is centered at this intermediate administrative level, allowing priorities, procurement, and implementation approaches to be aligned across the subnational health system.

By contrast, England operates at a much larger scale, administered by the UK’s national government and parliamentary framework even though health policy and service delivery in Scotland, Wales, and Northern Ireland are separate, governed by their specific devolved administrations. Differences in administrative scale are likely to influence which forms of system-level coordination are practical and achievable.

Within each site, we purposefully sampled individual participants to identify a diverse range of stakeholders who had been involved in conceiving, implementing, adopting and/or evaluating AI as part of system-wide interventions. We established initial contacts with key gatekeepers who facilitated contact with potential participants. These included academics, strategic decision-makers and policymakers with national/regional roles. We also used snowball sampling techniques asking each interviewee to recommend further participants. We sought to sample for a variety of backgrounds (strategic, academic, policy, technology), locations (geographical spread across the case study), and health-care settings (secondary, primary and community care).

### Data collection

Data were collected between April 2025 and March 2026. At each site, this involved analysis of documentary sources alongside semistructured, in-depth one-to-one online interviews and focus groups designed to capture perspectives on the rationale for, experiences of, challenges associated with, and lessons arising from system-level implementation of AI. Each case study was led by a designated member of the research team, who led on data collection and analysis.

Interviews and focus groups were conducted with topic guides exploring views on evolving strategies and needs, rationales for change, features of implementation and scaling approaches and technologies, expected and experienced benefits, experiences, lessons learnt, perceived challenges, and potentially transferable insights (Table 2).

**Table 2.** Sample topic guide.

Interviewee background Key features of health system organization and governance Background and history of digital transformation and AI in healthcare Overall AI strategy (past, present, and future) How AI in health is funded and role of government within this Approach to governance Approaches to data sharing and infrastructures Plans for and experiences of scaling Approaches to risk Monitoring and evaluation approaches Approaches to technology assessment and identifying technologies that can scale Approaches to partnerships and collaboration Overall lessons identified and potentially transferable aspects to NHS England

Abbreviation: AI, artificial intelligence.

Three focus groups (Norway, Catalonia, and Queensland) also included NHS England stakeholders to facilitate comparison, support the interpretation of findings, and explore transferability to the English context. NHS England was therefore not treated as a formal case study and was not subject to equivalent documentary analysis or interview data collection. The focus groups lasted between 2 and 2.5 h. Interviews lasted between 45 min and 1.5 h each.

Interviews and focus groups were recorded and transcribed verbatim by Microsoft Teams and subsequently reviewed for accuracy. We complemented interview and focus group data by collecting relevant local documents, including strategy documents, business cases, detailed implementation plans, benefits realization documents, lessons identified, press releases, and annual reports. We also collected documentation on the health system’s technological infrastructure, hardware, and the range of applications being implemented. Documents were mainly obtained through web searches and were publicly available.

After drafting narrative accounts of each case study, we shared draft findings with participants for comment. This process led to a small number of mainly descriptive clarifications.

### Analysis

We uploaded documents and transcripts into the qualitative research software NVivo and used the Technology, People, Organizations, and Macroenvironmental factors (TPOM) framework as a coding framework.^39^ We paid particular attention to emerging codes that did not fit in with the framework, those that spanned TPOM dimensions, and surprising findings. We analyzed data sources (documents and transcripts) within cases initially using a common data extraction sheet (Table 3). Comparative findings were then tested in focus group discussions. Data were coded by case study leads, who subsequently presented their findings to the larger team. Overarching themes were developed through discussions with all authors. The lead author synthesized the final analysis, with all authors contributing to interpretation and revisions.

**Table 3.** Characteristics of the 4 case study health systems.

Setting	Population (approx.)	Approximate health spend per capita^40^^,^^41^	Governance model	Degree of centralization	Funding approach	Digital infrastructure	Digital maturity	Relationship between AI and existing digital strategy	AI strategic orientation	Key scaling mechanism
Singapore	6.0 million	∼US $4000 (∼1000 voluntary/out-of-pocket)	National health system organized into 3 regional clusters	High	National innovation funding, health budgets, startup ecosystem, competitive grants	Epic-based infrastructure, national cloud environment, strong national AI platforms and research infrastructure	High	AI aligned with long-standing national digitalization and Smart Nation initiatives	Dual focus: health-care improvement and economic development through AI innovation	Bottom-up innovation with top-down orchestration and central scaling
Norway	5.5 million	∼US $8000 (∼1400 voluntary/out-of-pocket)	National system with regional health authorities	Moderate-high	Primarily core health system funding	Predominantly homegrown systems (DIPS), PACS infrastructure, regional cloud services, shared IT provision	High	AI embedded within existing national digital health strategy	Service improvement and coordinated adoption of market-ready AI	Regional coordination, shared procurement, platform-based deployment
Catalonia	8.0 million	Spain: US $4000 (∼1300 voluntary/out-of-pocket)	Autonomous regional health system within Spain	Moderate	European Union funding, regional public funding, innovation programs	Shared primary care EHR, openEHR architecture, central imaging archives, regional data spaces, cloud infrastructure	High	AI positioned as extension of regional digital transformation	Needs-based innovation with growing economic and industrial focus	Central validation, interoperability, coordinated diffusion across providers
Queensland	5.5 million	Australia: ∼US $7000 (∼1100 voluntary/out-of-pocket)	Federated state health system within Australia	Moderate-low	Mixed provider, regional and research funding	Statewide Oracle Health (Cerner) platform, shared EHR, cloud infrastructure, restrictions on offshore data storage	High	AI incorporated into broader digital and innovation strategy	Dual focus: service improvement and development of domestic AI capability	Middle-out governance, local experimentation and ecosystem coordination

Abbreviations: AI, artificial intelligence; EHR, electronic health record; PACS, picture archiving and communication systems.

In the analysis, we paid particular attention to exploring diverse participant perspectives, technologies and contexts. Across cases, we identified overarching themes, similarities and differences, transferable lessons, and potential policy implications for NHS England.

The findings from the cross-case analysis are presented in the “Results” section, and implications are presented in the “Discussion” section of this manuscript.

## Results

We conducted 34 qualitative interviews, 5 focus groups and collected 60 documents across the 4 health systems (Table 4). We consulted a total of 50 different participants across data collection activities. Participants included executive and system strategy leaders (*n*: 16, 5 from Catalonia, 4 from Queensland, 2 from Norway, 5 from Singapore); clinical leadership and clinical innovation (*n*: 14, 1 from Catalonia, 6 from Queensland, 2 from Norway, 5 from Singapore); AI/digital program and implementation leaders (*n*: 7, 3 from Catalonia, 2 from Queensland, 2 from Singapore); policy, evaluation and governance experts (*n*: 7, 3 from Catalonia, 1 from Queensland, 1 from Norway, 2 from Singapore); and technical, data, and research leadership (*n*: 6, 2 from Catalonia, 2 from Queensland, 2 from Singapore).

**Table 4.** Overview of the dataset.

Health system	Catalonia	Norway	Queensland	Singapore
Number of interviews	7	8	12	8
Number of documents	13	22	15	10
Focus groups	1 (8 participants from the Catalan case study and 3 from NHS England)	1 (8 participants, 4 from Norway and 4 from NHS England)	1 (7 participants, 4 from Queensland and 3 from NHS England)	3 (11 participants from Singapore)

We used the TPOM framework as a deductive coding framework to guide data extraction and analysis across case studies. However, rather than presenting findings according to the individual TPOM domains, the final thematic structure synthesizes findings across these domains to better reflect the interconnected nature of AI scaling within health systems. As shown in Table 5, each theme draws on multiple TPOM domains, illustrating how technological, organizational, professional, and macroenvironmental factors interact to shape system-level AI implementation and scaling.

**Table 5.** Overview of findings.

Theme	Subthemes	Principal TPOM domains
Existing digital infrastructure and institutional context	Strategy, leadership and governance Funding structures Technological infrastructures	Technology, Organization, Macroenvironment
Governance mechanisms for coordinating AI scaling	Multilevel orchestration in a polycentric system characterize current efforts to coordinate scaling of AI Systems must balance between competition and alignment Elements of coordination of scaling influenced the way scaling was coordinated	Organization, People, Macroenvironment
Fostering sustained use and spread in an innovative ecosystem	Evaluation and post-market surveillance Learning and collaboration Procurement as a key lever	Technology, People, Organization, Macroenvironment

Abbreviations: AI, artificial intelligence; TPOM, Technology, People, Organization, Macroenvironment.

It is important, however, to interpret comparative analysis with caution. Health systems are highly complex organizational entities that differ markedly in their historical development, organisational structures, funding and governance models, technological infrastructures, and existing digital estates. These contextual variations shape both the challenges encountered and the range of strategies available for deploying AI, limiting the scope for direct comparison.

The cases were purposively selected and represented health systems that had given substantial consideration to the practical challenges of AI implementation and scaling. This focus enabled detailed exploration of emerging approaches and lessons learned, although it may also mean that the findings do not fully reflect the experiences of all systems. While the United States has seen a number of large-scale AI deployments in healthcare, it was not selected as a case study because its health-care system differs substantially from the NHS in terms of funding, governance, procurement, and digital infrastructure. The US health-care landscape is highly fragmented, with multiple payers and providers operating under different incentives and regulatory frameworks. Consequently, factors that have enabled AI adoption at scale in some US organizations may not be readily transferable to publicly funded, nationally coordinated health systems.

Nonetheless, by examining diverse settings facing similar pressures, we identified several cross-cutting themes and processes that appear to support progress in the safe and effective deployment of AI across regions and health systems. These insights should therefore be viewed as analytically transferable rather than directly generalizable, offering guidance for policy and practice while remaining sensitive to contextual differences. There is no simple or universal relationship between change strategies and outcomes: approaches that are effective in one context may not translate readily to another.

Table 5 and Figure 1 provide an overview of findings, which we will discuss further in the paragraphs below. The findings are organized around 3 interrelated themes that describe how health systems approach AI scaling. First, existing digital infrastructures, governance arrangements, and institutional contexts establish the conditions under which AI can be developed and deployed. Second, health systems coordinate scaling through processes of multilevel orchestration that balance local innovation with system-wide alignment. Third, sustained use and wider spread depend on evaluation, learning, procurement, and collaboration within a broader innovative ecosystem. These themes are interdependent.

**Figure 1.**
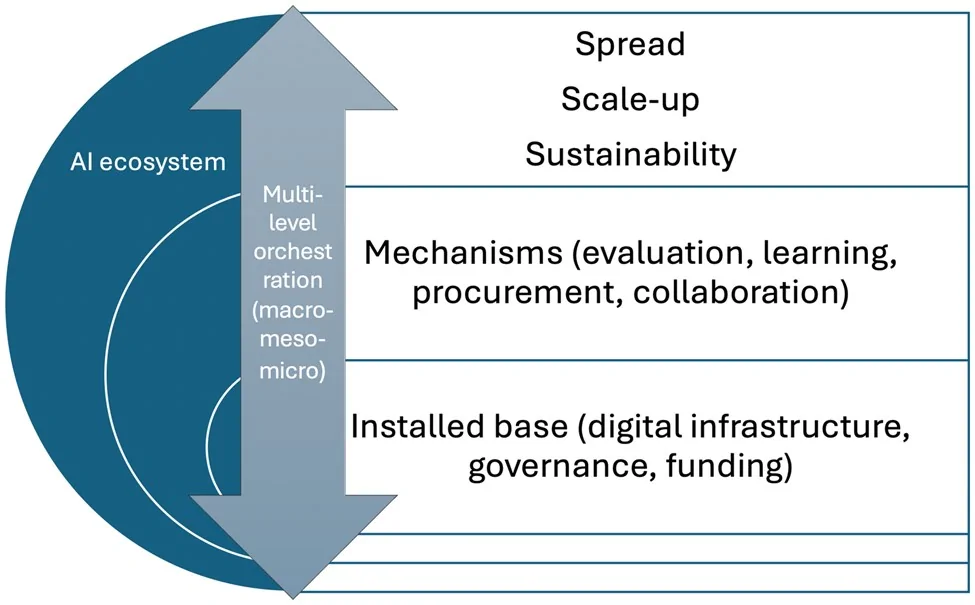
Overview of findings.

Across all 4 case studies, AI activity in health and social care was characterized by numerous pilots and demonstration projects but limited system-wide scaling, suggesting that health systems remain at early stages of the AI scaling journey. Applications included clinical applications such as radiology and pathology image interpretation, operational applications aimed at supporting capacity planning and patient flow, and more recent deployments of ambient clinical documentation and administrative automation tools. In all settings, AI was framed as a strategic priority and as part of broader digital transformation and economic growth agendas. At the same time, some stakeholders explicitly cautioned against hype and emphasized the need for a more measured and evidence-informed approach to deployment.

“We shouldn’t implement AI because it’s AI and because it’s cool, but because it gives you benefits.” Senior Decision-Maker, Norway

### Existing digital infrastructure and institutional context

Scaling trajectories were shaped by interrelated sociotechnical and sociopolitical contexts, including histories of digitalization, governance arrangements, leadership and strategy, procurement practices, technological infrastructures, and funding structures. Each case had developed a system-level AI strategy or strategic programme.

Although each jurisdiction studied had developed explicit AI initiatives, none described AI as representing a fundamental departure from existing digital transformation strategies. Rather, AI was consistently framed as building on established investments in digital infrastructure, governance, interoperability, and organizational capability. Differences between sites related primarily to the degree of centralization, leadership arrangements, and implementation mechanisms, rather than to the underlying strategic positioning of AI.

#### Strategy, leadership, and governance

Across health systems, common features of approaches to scaling included close working relationships between national/regional bodies and provider organizations. All health systems studied had a long history of pursuing digital transformation in healthcare, supported by relatively stable organizational arrangements and sustained leadership. In these contexts, AI was understood as an extension of an existing strategic trajectory rather than a discrete technology diffusion program, and there had been a strong and sustained focus on developing digital infrastructures. All 4 health systems shared an explicit ambition for system-wide deployment of AI as part of routine practice. Scaling was therefore considered from the outset, typically initiated within narrowly defined organizational or geographic boundaries followed by expansion (although these inevitably varied across health system settings).

In Norway, this ambition was operationalized through a deliberate strategy of creating or testing solutions locally and scaling those that demonstrated value. Concerted procurement played an important role: once contracts were established, hospitals outside the original procuring region could join, even if they already held different contracts. Particular sites were designated to progress early and then act as focal points for transferring experience and knowledge to others. Other sites were encouraged but not compelled to follow suit. Norway viewed AI primarily as a way of improving healthcare, while some of the other health systems studied had a dual track approach that also included wider economic goals to build homegrown AI capabilities. In Catalonia, Singapore, and Queensland, there was for example an explicit focus on economic benefit, on building domestic AI capability and on data sovereignty.

Queensland had a more informal scaling strategy than the other health systems studied. Here, local hospital and health services (HHSs) operated with a high degree of autonomy within a federal governance structure in which the national government was primarily responsible for policy and funding, while states managed and delivered health services. This arrangement meant that Queensland managed its own health system, within which HHSs had significant operational independence. The structure enabled imaginative, locally driven innovation and experimentation. With few formal methods for system-wide coordination, informal mechanisms of alignment, and learning prevailed, with some universities and hospitals exercising influence through their expertise and achievements. While there were both state-level and national ambitions to expand the use of AI, stakeholders expressed concern that the underlying digital and data infrastructure was not yet sufficiently developed to support large-scale, coordinated deployment.

“Governing and rolling out and deploying these solutions…could probably do some top down and go, this is it. You’re all gonna use this tool and this is where the data’s coming from and that guy’s responsible. But we don’t have that culture here.” Manager, Queensland

#### Funding structures

Funding arrangements differed markedly across the 4 settings and shaped the strategic direction of system-wide AI scaling. Singapore combined startup innovation funds, national health budgets, and regional innovation initiatives, reflecting a strong and explicit national commitment to creating an innovation economy.

“Generally, the projects are funded through competitive grants from the Ministry of Health or the National Medical Research Council.” Consultant, Singapore

In Norway, AI activity was largely supported through core health system funding. There was research-led innovation, but this played a relatively limited role in health system AI strategy. Catalonia had developed an active innovation cluster, largely oriented toward service improvement within the regional health system. While funding for research and innovation had often relied on European Union programs (reflecting persistent difficulties in securing central Spanish government support), recent efforts had increasingly focused on leveraging the region’s maturing digital and information-sharing infrastructure. This infrastructure enabled more systematic development, testing, and diffusion of AI and other digital tools across the Catalan health system. Although Catalonia had historically led on digital health service integration, a more explicit commercial and economic orientation recently emerged.

“The AI strategy is not only solving health problems… another objective as important as this one is to create and consolidate a technological industrial business fabric in Catalonia, helping the companies and the technological service environment of Catalonia to create solutions.” Senior Policymaker, Catalonia

In Queensland, AI initiatives drew on a range of funding sources, including provider budgets, regional funding mechanisms, and research grants.

“…eHealth Queensland…they’ve got a big bucket of money because they run the system, they run the platforms, all right. So that’s where the money often comes from. We’ve got some money from various other research sources, but nevertheless we also have had some funding from eHealth Queensland.” Clinician, Queensland

Across all health systems examined, there was a strong emphasis on clinical and medical research. In some contexts, this extended to structured efforts to support AI research, development, and commercialization. Singapore, and more recently Catalonia, had established national or system-level initiatives to promote AI innovation and industry collaboration, reflecting a strategic interest in developing and scaling new digital health technologies.

However, in all settings, the deployment of AI within health-care services was primarily service-led, with adoption typically driven by the goal of improving care delivery and operational performance. Norway represented the clearest case where service improvement was the dominant driver of AI activity. Singapore reflected a longer-standing tradition of state-led investment in research and innovation, but stakeholders emphasized that technologies needed to ultimately demonstrate practical value within health-care delivery.

At this early stage of applying AI in healthcare, a key driver was vendor and clinical enthusiasm to develop clinical AI applications particularly in areas such as radiology, pathology, and image-based diagnostics where data infrastructures and evidence bases are relatively mature.^4^ By contrast, relatively little attention across health systems was directed toward operational and pathway redesign applications, such as patient flow optimization, demand forecasting, and care coordination.

#### Technological infrastructures

Technological infrastructures to support AI differed substantially across the cases. Queensland and Singapore relied primarily on commercial platforms, whereas Norway and Catalonia had historically prioritized homegrown solutions. In Queensland, information governance rules and restrictions on offshoring Australian data had encouraged the use of domestically hosted clinical platforms and suppliers.

Across settings, electronic health records (EHRs) were understood primarily as data infrastructures. They were important for clinical research and for generating the data required to train and validate AI models. However, EHRs were not, in themselves, infrastructures specifically designed for AI deployment. While standardized EHR platforms could simplify technical integration and access to clinical data, their role in supporting large-scale model deployment and post-market surveillance was evolving. Major vendors were increasingly using their incumbent position within health systems to provide pathways for integrating AI tools directly into clinical workflows.

In all cases, the early deployment of clinical AI (including radiology image interpretation, fracture detection, pathology support, and other machine-vision-based diagnostic tools) was supported by preexisting infrastructures for storing and sharing medical images, most commonly through picture archiving and communication systems. These systems provided a mature and widely used foundation for accessing and exchanging diagnostic images across clinical settings, creating favorable conditions for integrating AI tools into existing workflows. This capability was enabled by the digital imaging and communications in medicine standard, introduced in 1993, which supports the seamless exchange of medical imaging data between systems. The presence of this infrastructure helped enable early adoption, particularly in imaging specialties, where both the underlying data environment and the evidence base for diagnostic AI were relatively well developed.

All health systems operated shared IT provision arrangements and common data protection frameworks. Catalonia, Queensland, and Norway had adopted cloud infrastructures provided by third-party suppliers, removing the need for individual organizations to maintain local servers and reducing barriers to transferring solutions between sites. Singapore operated a national cloud environment.

“We use the CSP which is the cloud service provider, be it from AWS [Amazon Web Services] or Azure. It wasn’t what the commercial cloud provides you because we do have our own sets of security.” Senior Consultant, Singapore

These institutional characteristics shaped not only what AI could be implemented but also how health systems coordinated scaling activities across organizations.

### Governance mechanisms for coordinating AI scaling

The central coordination challenge identified across the case studies was how to organize decision-making, align priorities across multiple organizations, and support the transfer of knowledge and learning throughout the health system. Different governance arrangements, rooted in differing institutional contexts and historical trajectories, resulted in a range of coordination mechanisms operating across macro- meso-, and microlevels. The findings presented in this section therefore focused on how health systems coordinated AI scaling and the contextual factors that shaped the effectiveness of different approaches.

Despite the frequent dichotomy drawn between “top-down” and “bottom-up” coordination models, approaches to AI scaling did not correspond to either of these extremes. Systems sought to balance innovation and exploitation by selectively coordinating the scaling of solutions with demonstrable system-wide value, while allowing other developments to remain locally driven.

“Governance is always a balancing act of three different things. You have to balance the political top-down governance. You have to balance and also have the bottom-up governance, the interest of all the different stakeholders, but there’s also the market governance…” Senior Policy-Maker, Norway

Scaling efforts took the shape of multilevel orchestration in a polycentric system of many stakeholders with various interests and priorities.^21^^,^^22^ We use the term “orchestration” to refer to activities aimed at coordinating, aligning, and guiding diverse stakeholders and resources toward shared goals. Orchestration was characterized by diverse sources of power and spheres of influence attempting to manage and govern AI. The orchestration challenge at the system level thus involved coordinating divergent interests and working across macro- (national policy and regulation, markets), meso- (organizational and network-level governance), and microlevels (clinical and operational). There was a broad recognition that most systems initially experienced uncoordinated local experimentation, as individuals and specific organizations explored potential AI applications. While this “bottom-up” activity enabled early learning, it often proved slow and costly, with duplicated procurement, validation, and governance processes and persistent interoperability challenges. Historically, weak coordination associated with “bottom-up” experimentation had resulted in highly heterogeneous technological and organizational environments, thereby increasing the costs of alignment and orchestration. At the same time, stakeholders noted that purely “top-down” approaches struggled to respond effectively to the heterogeneity of local contexts and needs, including clinical workflows, institutional legacies, and infrastructural constraints. As a result, many systems were seeking more balanced approaches that combined local experimentation with stronger mechanisms for coordination and shared learning.

“What is needed is this governance in which it is clear which actors must participate, because otherwise they keep duplicating [efforts]… It’s about trying to 'put in order’ how a technology that demonstrates value enters the system… to guide the process and avoid losing opportunities and avoid duplicities. It is trying to build a path from whoever promotes innovation until whoever really helps with adoption.” Health Technology Assessment, Catalonia

Multilevel orchestration in a polycentric system therefore best described these attempts to coordinate scaling of AI across health systems. Although approaches varied in form, they shared a common premise: systems sought to balance competition and alignment.

Within this context, we observed several common elements that shaped how scaling was coordinated in a polycentric environment. First, as outlined above, infrastructure and the installed base of technologies and organizational arrangements strongly influenced coordination. In Singapore, cluster-level platforms linked to core clinical systems allowed local innovation while maintaining shared data infrastructures.

“So, Endeavor AI is the platform that is a production grade AI platform that’s connected to our EMR [Electronic Medical Record] system… We extract data live out of Epic in real time from the engine.” Senior Consultant, Singapore

In Queensland, large commercial platforms introduced some elements of “top-down” coordination, while parallel “bottom-up” innovation continued to occur. The state was also developing a shared data infrastructure outside its core vendor platform to avoid vendor control over data integration.

“…we will purchase packaged software solutions where they will run artificial intelligence over the data that we put in it, but then we’ll want to take that data and mash it up with other data that’s in a different box with a different algorithm, and we might want to do something with that superset. And we don’t have any answer for that other than our own platform with our own data scientists and things like that.” Manager, Queensland

Second, temporality played a critical role in coordination. We examined homologous learning processes across sites unfolding incrementally under changing circumstances. The incentives and processes that shaped innovation operated differently at different stages of development. Early phases tended to emphasize diversity and experimentation, allowing multiple approaches and solutions to emerge through local initiatives and pilot projects. Over time, however, this diversity gave way to more selective support for options that demonstrated effectiveness in practice. Systems therefore moved gradually from experimentation toward coordination, with increasing efforts to align organizations around solutions that had proven successful. Throughout the process, there was a balance between diversity and competition on the one hand, and coordination, selection, and alignment on the other hand, but the emphasis typically shifted toward alignment as technologies matured and experience accumulated. Early experimentation was typically conducted at small scale within specific local settings. As systems moved toward wider adoption, central or regional decision-making became more prominent. For example, in Singapore, experimentation was followed by explicit “top-down” decisions about which innovations would be scaled nationally, and which would remain regional or be discontinued. In Norway, dissatisfaction with earlier large-scale digitalization programs had led to the development of a more intricate coordination mechanism. Nationally agreed priorities helped guide technology uptake, while certain provider organizations were designated as exemplars to test and implement solutions. Through contractual arrangements, other providers could then adopt tools and benefit from the experience and procurement arrangements established by the exemplar sites. In Queensland, by contrast, coordination remained relatively informal. As in Norway, coordination partly occurred through exemplar organizations, but the mechanisms differed. In Norway, regional funding supported selected sites to lead innovation and then disseminate experience to others. In Queensland, leadership tended to emerge more informally, with certain organizations gaining prominence through successful innovation initiatives or funding competitions. In each case, wider alignment depended heavily on trust in exemplar sites and the credibility of their experience, rather than on direct mandate or coercion.

Third, incentives shaped alignment. Professional recognition, organizational status, control over infrastructure, and the perceived benefits of either conforming to shared solutions or pursuing independent approaches all influenced whether organizations chose to align with wider system strategies. When working well, this alignment was often less the result of a formal mandate and more a product of trust. Provider organizations were willing to treat the experience of exemplar sites as credible and relevant to their own context, allowing learning to diffuse through informal recognition rather than through coercion or central directive. Incentives therefore mattered, but alignment tended to emerge through confidence in trusted peers rather than through imposed compliance.

“We have also decided to allocate money to them for three years in a row to help the other hospitals… instead of having a regional project or the IT service provider or us for that matter, telling them what to do, we have a more sort of a peer-like another hospital, another clinic with more experience telling these hospitals what to do…” Senior Decision-Maker, Norway

Fourth, coordination operated across multiple decision spaces: central, regional, and local. Some health systems had developed explicit and evolving structures to link these decision levels and manage interdependencies. Here, we observed 2 distinct patterns. In Singapore, Norway, and Catalonia, strong central oversight was combined with structured local innovation. Infrastructure capable of supporting deployment across sites was essential, and early successes were identified and actively pushed throughout the system. Local experience became the basis for wider uptake through 2 main mechanisms: knowledge flows, where learning was shared across organizations, and alignment, where technological, governance, and cultural configurations were stabilized and reproduced across sites to avoid duplication and reduce uncertainty.

A contrasting pattern was characterized by predominantly localized activity with limited central coordination. Queensland most closely reflected this configuration. High organizational autonomy had constrained regional alignment and shared learning, and stakeholders emphasized the need to establish stronger coordination structures, potentially linked to research rather than service delivery funding.

“So, in Australia what’s happening is it’s mostly driven from your research institutions. So, your universities, they have researchers, professors, you know, fellows that want to do computer science, work with epidemiology, work with whoever to do AI on whatever. Public health is a big one because of big data.” Clinical Researcher, Queensland

However, although we observed common patterns in coordination, these did not always apply. More recently, radical “bottom-up,” user-driven forms of scaling had emerged, for example with the proliferation of ambient clinical documentation tools that automatically generate clinical notes and administrative records from clinician-patient interactions. These differed substantially from earlier generations of AI applications, such as radiology and pathology decision-support systems, which had shaped existing governance and scaling strategies.

Coordination mechanisms alone were insufficient to support sustained AI scaling. Across case studies, effective coordination depended on complementary processes for evaluation, procurement, learning, and collaboration, which are discussed in the following section.

### Fostering sustained use and spread in an innovative ecosystem

We found that multilevel coordination could help to foster sustained use and spread but these efforts should be framed within wider efforts to build an innovative ecosystem. In Singapore, many AI activities were coordinated through regional health-care clusters that brought together multiple hospitals, community providers, and supporting organizations. Several interviewees held cluster-level leadership roles responsible for coordinating strategy, innovation, and operational activities across these health-care networks.

“For us the one of the key ways that we look at scaling is that when we start working on AI, we have to think of the ecosystem in which we are going to implement it because quite often there’s a lot of focus on the product, but not enough focus on the processes and the ecosystem that will allow it to flourish.” Cluster Manager, Singapore

#### Evaluation and post-market surveillance

The ability of AI systems to remain effective and relevant over time depended on ongoing monitoring after deployment, including workflow optimization, maintenance, and system updates. Because long-term sustainability remained uncertain, investment decisions needed to take account of short-, medium- and long-term horizons. This uncertainty reinforced the central importance of evaluation and learning in supporting sustained adoption across contexts.

Current evaluation practices across health systems were diverse and include regulatory assessments, local clinical governance processes, and post-market surveillance. However, these approaches varied in depth and methodological rigor, and there was increasing recognition of the need for systematic validation of tools and for the development of appropriate evaluation methods with attention to model drift, safety and unintended consequences.

Nationally and regionally coordinated approaches to evaluation remained limited. Catalonia was a notable exception. Here, evaluation was positioned as a national service for delivery teams. The Catalan case also emphasized a shift from one-off approval to lifecycle stewardship, evaluating whether tools remained safe, fair and effective in routine use and how to intervene if performance changed. However, these structures were still in the early stages of development.

Balancing risk assessment with potential clinical and operational impact remained a persistent challenge. In Queensland and Singapore, many low-risk deployments were decided locally with explicit permissive strategies of governing low-risk systems.

“Low-risk AI (e.g., administrative automation) remains under local governance with self-assessment tools.” Queensland Health’s AI governance framework

#### Learning and collaboration

Stakeholders also emphasized the need to align incentives across organizations, promote collaboration, and invest in AI literacy among clinical and managerial staff. Continuous learning and optimization were increasingly recognized as integral to scaling, linking national coordination with local improvement processes. Here, local exemplars played a key role in diffusing practice, and incentive mechanisms (such as Singapore’s multicluster funding) were used to promote collaboration. In Norway and Queensland, particular organizations were encouraged to take a lead in specific domains and to act as exemplars, with others invited to follow their approaches rather than being required to adopt identical solutions.

“We don’t want to do all do the same things all over again. But now we’re trying to trust each other. So, we say if we do this, you can do the same as we are doing.” Senior Decision-Maker, Norway

Artificial intelligence repositories and other low-cost mechanisms for sharing tools and experience were viewed as valuable self-coordination devices, supporting fluid circulation of expertise.

Strong links with universities and research organizations influenced the balance between innovation and learning. This was particularly evident in Queensland, where clinical research activity was prominent, and clinicians were often able to experiment with AI tools under research governance arrangements that provided basic ethics and governance arrangements. However, as tools moved from research settings into routine service delivery, governance, validation and commercialization requirements became substantially more demanding. Constraints on data exploitation further shaped sustainability pathways and tended to advantage domestically developed tools.

“I think you do have to, particularly when it gets to clinical cases. I think there have to be really strong partnerships with the university.” Clinician, Queensland

University partnerships supported the innovative ecosystem in 2 ways. First, they fostered learning communities that facilitated the exchange of expertise and experience. Second, they underpinned clinical research and local tool development. However, research-oriented clinicians and teaching hospitals sometimes prioritized studies to advance medical knowledge over deploying tools in everyday practice, and optimizing and exploiting them to transform care processes.

#### Procurement as a key lever

Finally, procurement was viewed as a key lever. However existing procurement models often struggled to manage AI as a changing service rather than a stable product. Countries were therefore actively developing new procurement models. Procurement practices were evolving to help manage uncertainty, build organizational capability, and reshape relationships with suppliers. For example, in Catalonia, there was increasing emphasis on approaches that supported learning and adaptation over time, rather than the one-off purchase of fixed products.

“We can’t buy AI as if it were a fixed product. What we purchase is a service that learns and changes with our data and workflows. That means validating on Catalan data before deployment, monitoring continuously for drift, bias, and safety, and encoding this in the contract, we must procure uncertainty, not just the product.” Health Innovation Strategist, Catalonia

Norway had gone further in experimenting with new procurement models. These included a novel “competitive dialogue” procurement process which led them to purchase AI models through platform-based arrangements and subsequently to procure multiple platforms to create an ecology in which model developers competed based on cost and performance. Novel, more open and flexible contractual arrangements also allowed multiple provider organizations to adopt solutions initially procured by designated exemplar sites.

“When we first did a procurement for the fracture application… now we did a new mini competition on these three platforms for the same application… the price was half.” Manager, Norway

Taken together and as illustrated in Figure 1, the findings suggest that AI scaling is best understood as an interaction between 3 interdependent elements: an established institutional and digital foundation; mechanisms for coordinating action across multiple actors and organizational levels; and an innovative ecosystem that supports learning, evaluation, procurement, and continuous adaptation.

## Discussion

### Summary of main findings

As illustrated in Figure 1, the case studies suggest that AI scaling is not a linear process but one shaped by interactions between existing institutional capabilities, mechanisms for multilevel coordination, and wider innovation ecosystems. Across the case studies, health systems had typically undergone a period of learning and adaptation. Early experience often exposed the limitations of both highly centralized strategies and more laissez-faire approaches based on uncoordinated local adoption. Over time, these systems had put in place more developed mechanisms to support shared learning and encourage more coordinated patterns of uptake. While the specific arrangements varied according to differences in institutional structures, existing technological infrastructures, and policy contexts, clear commonalities nevertheless emerged across cases.

Two features appeared particularly significant. First, systems sought to strike a balance between competition and coordination, enabling diversity and experimentation in the early stages while progressively strengthening alignment around approaches that demonstrated value in practice. Second, they supported the emergence of trusted exemplar sites. These organizations acted as early adopters and sources of practical learning, with other providers choosing to align with their experience. Such alignment was typically fostered through informal leadership, professional credibility, and structured opportunities for knowledge sharing, rather than through strong central mandates.

The sustained use and wider spread of AI can be supported by continued monitoring following deployment, alongside the concerted collation and dissemination of emerging evidence. These activities need to be embedded within a broader innovative ecosystem that prioritizes learning, partnership working, governance, ongoing evaluation, and adaptive procurement approaches.

None of the jurisdictions studied viewed AI as requiring a fundamental departure from their existing digital health strategies. While the organizational arrangements, governance structures, and implementation mechanisms differed across settings, participants consistently described AI as building upon established investments in digital infrastructure, interoperability, governance, workforce capability, and innovation programs. The central strategic question therefore was how existing digital capabilities were mobilized and coordinated to support AI adoption and scaling. This finding suggests that health systems seeking to accelerate AI deployment may derive greater benefit from strengthening the institutional and digital foundations that support innovation than from developing AI-specific strategies in isolation.

### Strengths and limitations

We conducted a systematic qualitative comparative analysis of how 4 health systems approached the scaling of AI in health-care settings. Our participants consisted of senior stakeholders involved in orchestrating AI strategies at system level. This provided valuable insights into national and regional planning processes, coordination mechanisms, and policy rationales, offering a high-level overview of how competing stakeholder interests were negotiated in the early stages of scaling rapidly evolving technology.

The cases were selected because they appeared to have made notable progress in developing strategies to scale and coordinate the adoption of AI. This study was therefore not intended as a comprehensive survey of all health systems, and experiences from other settings are also likely to provide important insights. The United States was not included in the sample. Although it represents a globally important setting for AI innovation and deployment, its more fragmented governance arrangements differ substantially from the system-level approaches examined here and warrant dedicated future study. In addition, alternative models may emerge over time. There is consequently scope to extend this analysis both longitudinally, as current approaches continue to evolve, and geographically, through the inclusion of additional systems and contexts.

A key strength of this work was its cross-national design, which enabled the identification of both shared patterns and important contextual differences in AI scaling strategies across health systems with diverse demographic characteristics, governance arrangements, and service configurations. Despite this heterogeneity, the analysis revealed significant similarities in strategic responses and implementation challenges, suggesting that common underlying factors may be shaping the adoption and spread of AI internationally.

However, there were also several limitations. Recruitment of sites proved challenging, and participation was largely limited to settings where existing contacts with key gatekeepers facilitated access, as well as those organizations that responded positively to our invitations. As a result, the sample may overrepresent health systems that were willing or able to showcase their strategies. Participants may inevitably have tended to emphasize successes and positive developments, with a relative reluctance to discuss initiatives that had not progressed as intended. This is understandable given that many interviewees occupied senior policy or strategic roles and may have focused more on achieving desired outcomes and barriers overcome than on highlighting negative experiences. Continued exploration of these cases with a broader range of stakeholders would help achieve a more robust and comprehensive account of these developments as well as lessons emerging from these initiatives.

We also primarily captured the perspectives of actors responsible for coordinating and overseeing AI initiatives. Frontline health-care staff, commercial stakeholders, and patients were not included, meaning that our findings reflect planned strategies and intended trajectories rather than how AI systems and strategies were experienced, interpreted, or adapted in everyday practice. This reflects the polycentric nature of the system, illustrating that we obtained access to some parts of polycentric system but not to others.

Finally, participating health systems were at relatively early stages of their AI scaling journeys reflecting the wider immaturity of the sector. Our analysis therefore reflects anticipatory strategies rather than medium- or long-term outcomes. Narratives were not independently validated through fieldwork within health systems, relying instead on participant accounts and documentary analysis. While trust developed over the course of the study and participants were invited to review case studies and the final report (resulting in minor clarifications) future longitudinal research will be important to assess how these strategies unfold in practice over time.

### Integration of the findings with the existing literature

The conceptual framework presented in Figure 1 shows that successful scaling depends on the alignment between institutional capabilities, coordination mechanisms, and continuous learning processes operating across multiple organizational levels.

Our findings reinforce existing accounts that conceptualize AI scaling in healthcare not as a linear process, but as an adaptive, contextually situated activity that requires active orchestration.^21–23^ Rather than progressing through a series of predictable stages, AI scaling unfolds through the iterative configuration of technology, infrastructures, relationships, organizations, and wider system environments.

This perspective aligns with the policy learning and learning health systems literature, which emphasizes the role of infrastructures, socio-organizational contexts, and ongoing governance and learning mechanisms in enabling system change.^16–20^^,^^23–25^ Our work adds that scaling trajectories are shaped not only by existing strategy but also by historical health system contexts that together form the installed base, which shapes the range of pathways through which scale can be pursued.^42^ These pathways are influenced not only by historical development trajectories and coordination efforts but also by existing health system configurations, technological infrastructures, strategic priorities, and organizational cultures.

Our findings challenge conventional framings that position scaling as a choice between “top-down” and “bottom-up” coordination strategies.^43^^,^^44^ Power and agency in AI development and deployment are distributed across multiple actors, with overlapping and sometimes competing interests. AI scaling is therefore more accurately understood as occurring within polycentric systems, where influence is dispersed across stakeholders operating at different levels.^45–47^ In this context, the core challenge shifts from one of implementation to one of multilevel orchestration, requiring continuous negotiation as stakeholder interests and capabilities evolve and technological potential and development change within the constraints of the local installed base. Here, scaling should not simply be viewed as an expression of power driven by political or commercial pressure for rapid expansion.^48^ Instead, the experiences examined suggest the importance of carefully balancing strategies between competition and cooperation, allowing experimentation and diversity in early stages while progressively encouraging greater alignment as effective solutions emerge.

A further common feature is the strongly informal character of many coordination mechanisms. Rather than relying on highly formal approaches, adoption is typically voluntary, supported by financial incentives, shared learning, and relationships of trust, often reinforced through partnerships and learning. These coordination arrangements are embedded within a broader multilevel ecosystem in which factors at macroenvironmental, interorganizational, organizational, and individual levels collectively shape how collaborations emerge and evolve.^49^

At the macrolevel, policy developments, program mandates, technology supplier strategies, and intermediary actions shape the broader conditions for collaboration. At the interorganizational level, relational recognition, collective identity, governance arrangements, and coordination practices influence how partnerships are formed and sustained. Within organizations, capabilities such as external search, absorptive capacity, prior collaboration experience, and internal knowledge routines affect their ability to engage with these networks. At the individual level, participation is shaped by actors’ motivations as well as potential personal barriers. Taken together, these multilevel dynamics contribute to the formation of a learning ecosystem in which relationships evolve along a continuum between formal and informal arrangements and between internally and externally initiated collaborations, enabling diverse forms of coordination and knowledge exchange across the system.

Addressing this multilevel orchestration challenge requires approaches that support learning and coordination over time without being overly prescriptive and adapting to changing circumstances and needs.^50^ Creating an enabling innovative ecosystem is critical in this context.^51^ Here, scaling must be conceptualized as a process of sustained system-level transformation rather than a discrete act of technology implementation.^52^ Our study suggests that AI scaling in healthcare requires a distinct focus on learning and evaluation. This should include embedding evaluation and evidence generation across the AI lifecycle and linking these insights to strategic decision-making; creating mechanisms that connect stakeholders across organizational and sectoral boundaries to aggregate learning from pilots and support communities of practice; and reconceptualizing governance and procurement frameworks to better respond to the emergent risks, responsibilities, and coordination demands associated with AI.

A notable finding across case studies was the increasing shift from one-off technology assessment toward continuous evaluation throughout the AI lifecycle. Rather than treating implementation as the endpoint, several health systems were beginning to establish mechanisms for post-deployment monitoring, surveillance for model drift, bias assessment, safety monitoring, and benefit realization. This represents an important transition from static evaluation of software products toward stewardship of adaptive technologies. Developing national or regional evaluation infrastructures may therefore become as important as investment in AI technologies themselves. Shared evaluation services could reduce duplication, support procurement decisions, facilitate evidence generation across organizations, and provide early warning of deteriorating performance.

### Recommendations for policy and practice

As illustrated in Figure 1, interventions targeting only one element of the system (eg, procurement or governance alone) are unlikely to achieve sustainable scaling unless supported by appropriate institutional capabilities and mechanisms for learning and coordination.

If scaling AI in health systems is understood as a multilevel orchestration challenge within a polycentric ecosystem, then policy and practice need to move beyond linear “adopt and spread” models toward approaches that acknowledge fragmentation, shifting power dynamics, temporal changes and tensions, and the inherently evolving nature of AI systems. Efforts therefore must balance encouraging local experimentation and aligning a fragmented multilevel array of stakeholders across regional and national bodies, provider organizations, suppliers, and the public. Without coordination there is a risk of duplication, uneven governance, and variable evidence standards. Policy must therefore distinguish between what requires central coordination (such as safety standards, equity safeguards, interoperability, and evidence expectations) from areas where local adaptation and learning should be protected. Local autonomy remains crucial for innovation, particularly in organizations with digital capability and risk appetite, but national or regional coordination is necessary to support evaluation, sharing of learning, and equity.

Although the cases examined were predominantly publicly funded health systems, with relatively coordinated governance arrangements, many of the challenges identified are likely to be even more pronounced in more fragmented health-care environments characterized by distributed governance, mixed public-private provision, and multiple purchasing organizations. In such settings, balancing local innovation with system-wide scaling becomes more difficult because procurement, digital infrastructure, and implementation decisions are distributed across multiple independent organizations rather than coordinated centrally.

Our findings suggest that the mechanisms identified in this study (including shared approaches to evaluation, post-market surveillance, evidence generation, procurement, interoperability standards, and knowledge exchange) may provide important ways of supporting AI scaling where central authority is limited. Rather than relying primarily on top-down coordination, fragmented systems may need stronger regional collaborations, intermediary organizations, and shared governance arrangements that facilitate learning and coordination across organizational boundaries while preserving local autonomy. The concept of multilevel orchestration, supported by shared approaches to evaluation and organizational learning, therefore provides a useful way of thinking about AI scaling across a wide range of health-care systems, irrespective of their funding or governance arrangements. Although institutional arrangements will differ, the need to coordinate innovation, evaluation, procurement, and digital infrastructure appears to be common across health-care systems.

In this context, orchestration activity must involve building in opportunities for iteration and learning over time as processes unfold whilst also cultivating a sustainable innovative ecosystem. This requires dedicated, sustained funding not only for technologies but also for the human and organizational infrastructure needed to monitor, curate, maintain, and update AI systems. It will also require investing in AI literacy, regulatory, ethical and procurement frameworks, areas that are currently still evolving. Policy should clarify shared stewardship arrangements between providers and suppliers and support standardized access to information on training data, bias testing, limitations, and update cycles to enable comparison and safe adoption.

Evaluation must inform these activities in real time as a shared, system-level responsibility. Whilst strong model accuracy is important, it does not guarantee adoption and effective integration with local settings. There is therefore a need for more systematic evaluation of AI in real-world settings, alongside mechanisms to collate, compare, and share emerging evidence.^53^ This implies a role for central or regional coordination in tracking implementations, supporting regional evaluation capacity, and maintaining registries of AI projects. Common evaluation frameworks can support comparability and learning across contexts, while mechanisms such as anonymous reporting of AI risks and failures may help surface issues that would otherwise remain hidden.^54^ As AI tools move rapidly from pilots to routine use, gaps in regulatory, implementation, and lifecycle literacy risk frontline teams inadvertently bypassing appropriate safeguards. Investing in tiered governance (“just enough governance”), alongside targeted training in regulatory thresholds, implementation risks, and post-deployment stewardship can help to address this issue. The aim should be not to slow innovation, but to make it safer and more scalable by ensuring clear escalation routes and accountability.

Many of the sites we studied experimented with new procurement approaches. Poorly specified procurement can shift uncertainty and risk onto adopting organizations. By contrast, procurement frameworks that incorporate requirements for transparency, system integration, ongoing monitoring, and lifecycle accountability can help shape safer and more effective markets. Procurement should therefore be aligned with evaluation expectations and used to secure appropriate supplier disclosure, rather than being treated as a purely transactional stage that follows innovation.

Finally, prioritization of what AI to scale when and where must be actively managed as an ongoing coordination task. Health systems are pulled between high-visibility, fast-moving market opportunities (such as ambient documentation tools) and slower, needs-led, pathway-based priorities grounded in service pressures. These logics reward different timelines and forms of progress. When visibility-led commitments dominate, scale can outpace governance and infrastructure. When pathway-led approaches dominate, momentum and investment may stall. Policy must therefore create mechanisms that balance speed with institutional readiness.

We summarize lessons for systemic change associated with scaling AI emerging from this work in Table 6.

**Table 6.** Lessons for systemic change associated with scaling AI in health-care settings.

Lesson for AI scaling	Why it matters	Effective approaches observed across cases	Contextual considerations
AI scaling should be viewed as system transformation rather than technology deployment	Successful adoption depended on changes to governance, workflows, evaluation, procurement, and learning systems, not simply introduction of AI tools	Development of innovation ecosystems incorporating governance, evaluation, procurement, workforce capability, and partnerships	Systems with fragmented governance may require stronger coordination mechanisms than more integrated systems
Balance local experimentation with system-wide coordination	Purely centralized and purely decentralized approaches both created challenges. Innovation benefited from local flexibility, while scale required alignment	Allowing organizations to pilot and test solutions while establishing common standards for safety, interoperability, and evaluation	The optimal balance will depend on existing governance structures, organizational autonomy, and digital maturity
Use exemplar organizations as engines of diffusion	Trusted early adopters generated practical implementation knowledge and reduced uncertainty for others	Supporting leading sites to evaluate, refine, and demonstrate AI use cases and share lessons through formal and informal networks	Exemplar models are most effective where there are mechanisms for knowledge transfer and professional credibility
Invest in learning infrastructure, not just technology	Scaling depended on the ability to continuously generate, interpret, and share evidence	Communities of practice, implementation networks, registries, evaluation programs, and shared evidence repositories	Resource-constrained systems may need regional or national support to sustain learning functions
Embed evaluation throughout the AI lifecycle	Technical performance alone did not predict successful adoption or impact	Continuous monitoring, post-deployment evaluation, real-world evidence generation, and feedback loops linking findings to decision-making	Evaluation frameworks should be proportionate to risk and adaptable to rapidly evolving technologies
Treat coordination as an ongoing orchestration challenge	AI scaling involved multiple actors with different incentives, capabilities, and priorities	Active management of relationships between policymakers, providers, suppliers, regulators, and professional groups	Polycentric systems may require greater emphasis on facilitation and consensus-building than command-and-control approaches
Develop governance that supports innovation while managing risk	Overly burdensome governance may slow adoption, while weak governance risks harm (eg, excessive diversity which is expensive and harder to govern for safety) and loss of trust	Clear safety standards, transparency requirements, accountability arrangements, and tiered (“just enough”) governance approaches	Governance arrangements should reflect organizational capability, AI risk level, and regulatory context
Align procurement with implementation and evaluation goals, and innovation dynamics	Procurement decisions shaped long-term safety, sustainability, and scalability	Procurement frameworks requiring transparency, interoperability, monitoring, supplier accountability, and lifecycle support	Procurement capability and market maturity vary considerably between health systems
Build workforce capability and AI literacy	Adoption depended on the ability of organizations and staff to understand, evaluate, and manage AI technologies	Investment in technical, clinical, implementation, regulatory, and procurement skills	Workforce needs differ according to existing digital maturity and local capability gaps
Prioritize AI deployment strategically rather than opportunistically	Health systems faced tensions between highly visible technologies and needs-driven service priorities	Structured prioritization processes balancing innovation opportunities with service needs, readiness, and governance capacity	Priorities should be aligned with local health system pressures, strategic objectives, and implementation capacity
Support adaptation rather than standardization alone	Effective AI implementation required tailoring technologies to local workflows and organizational contexts	Flexible implementation approaches combined with common expectations around evidence, safety, and interoperability	Greater adaptation may be needed in heterogeneous systems and across diverse care settings
Foster partnerships and trust across organizational boundaries	Knowledge exchange and coordinated action often depended more on relationships than formal mandates	Multiorganizational collaborations, learning networks, and trusted communities of practice	Trust-building may be particularly important where organizations compete for resources or operate under different incentives

Abbreviation: AI, artificial intelligence.

While governance arrangements differ considerably between health systems, investment in shared evaluation and learning infrastructures may represent a transferable strategy across a wide range of institutional settings.

## Conclusions

Scaling of medical AI must be understood not simply as implementation and deployment, but as a continuous process of adaptation, evaluation, and collective learning within a wider innovative ecosystem context. Orchestration of AI scaling therefore must be viewed as a multilevel process within a polycentric system that is neither “top-down” nor “bottom-up” but involves a careful balancing act between managing various stakeholder interests and spheres of influence. This requires strategies that can cope with uncertainty, are sensitive to context, and are capable of supporting both technological cultivation and system-level transformation. Robust approaches to evaluation and learning are essential components of this process, helping to guide effective coordination and maximize the potential for AI to improve health-care outcomes.

## Data Availability

The data underlying this article are not publicly available because they consist of qualitative interview transcripts and associated field materials containing potentially identifiable information. Participants did not provide consent for public data sharing, and sharing the data could compromise participant confidentiality. Deidentified excerpts supporting the findings are included within the article. Requests for additional information may be directed to the corresponding author and will be considered on a reasonable request basis, subject to institutional ethical approval and data sharing agreements.
